# Olfactomedin 4 deficiency promotes prostate neoplastic progression and is associated with upregulation of the hedgehog-signaling pathway

**DOI:** 10.1038/srep16974

**Published:** 2015-11-19

**Authors:** Hongzhen Li, Wenli Liu, Weiping Chen, Jianqiong Zhu, Chu-Xia Deng, Griffin P. Rodgers

**Affiliations:** 1 Molecular and Clinical Hematology Branch, National Heart, Lung, and Blood Institute, National Institutes of Health, Bethesda, MD 20892, USA; 2 Genomics Core Facility, National Institute of Diabetes and Digestive and Kidney Diseases, National Institutes of Health, Bethesda, MD 20892, USA; 3 Genetics of Development and Disease Branch, National Institute of Diabetes and Digestive and Kidney Diseases, National Institutes of Health, Bethesda, MD 20892, USA

## Abstract

Loss of olfactomedin 4 (*OLFM4*) gene expression is associated with the progression of human prostate cancer, but its role and the molecular mechanisms involved in this process have not been completely understood. In this study, we found that *Olfm4-*knockout mice developed prostatic intraepithelial neoplasia and prostatic adenocarcinoma. Importantly, we found that the hedgehog-signaling pathway was significantly upregulated in the *Olfm4*-knockout mouse model. We also found that restoration of *OLFM4* in human prostate-cancer cells that lack *OLFM4* expression significantly downregulated hedgehog signaling-pathway component expression. Furthermore, we demonstrated that the OLFM4 protein interacts with sonic hedgehog protein, as well as significantly inhibits GLI-reporter activity. Bioinformatic and immunohistochemistry analyses revealed that decreased *OLFM4* and increased *SHH* expression was significantly associated with advanced human prostate cancer. Thus, olfactomedin 4 appears to play a critical role in regulating progression of prostate cancer, and has potential as a new biomarker for prostate cancer.

Prostate cancer is the most commonly diagnosed solid tumor and the second-leading cause of cancer-related death in the American male population^1^. Alterations in specific gene products and molecular pathways often occur in prostate cells during prostate carcinogenesis and metastasis. It has been found that activation of epidermal growth factor receptor, sonic hedgehog, Wnt/β-catenin, and stromal cell-derived factor-1 (SDF-1)/CXC chemokine receptor 4 (CXCR4) occurs during prostate-cancer progression from locally invasive to metastatic and castration-resistant prostate cancer^2^. Hedgehog signaling mediates prostate ductal morphogenesis^3^, regeneration, neoplasia, and prostate-cancer cell metastasis^4^,^5^. In addition, hedgehog signaling is required for maintaining cancer stem cells in human mammary tumors^6^, myeloid leukemia^7^, and in airway epithelial progenitors and small-cell lung cancer^8^, as well as for regenerative proliferation in bladder epithelial stem cells^9^. Hedgehog overexpression has also been shown to induce prostate cancer in mice with *Shh*-expressing plasmid injected into the prostate^10^.

The human *OLFM4* gene (olfactomedin 4, also known as hGC-1, GW112, and hOlfD) was first cloned from human myeloid progenitor cells and encodes an OLFM4 protein that is normally expressed in prostate, bone marrow, small intestine, and pancreas^11^. OLFM4 plays an important role in prostate cancer^12^, gastrointestinal cancer^13^,^14^, and myeloid leukemia^15^. Frequent genetic deletion of the *OLFM4* gene has been found in advanced prostate cancer^16^ and squamous cell carcinomas^17^. Gene-expression data have revealed downregulation of the *OLFM4* gene in prostate cancer, colon cancer, and leukemia, whereas *OLFM4* expression was found to be upregulated in gastric cancer and pancreatic cancer. These divergent results may be due to tissue- and cell-specific factors, as well as inflammation status and tumor grade^18^.

In this study, we examined the potential functions of the *Olfm4* gene in murine prostate tissues by analyzing an *Olfm4*-knockout mouse model, and found that loss of *Olfm4* leads to neoplastic progression in the mouse prostate, as well as tumor formation in the lung, liver, and pancreas. We further explored the possible molecular mechanisms underlying prostate neoplastic progression in *Olfm4*-knockout mice and found that *Olfm4* negatively regulates the hedgehog-signaling pathway in mouse prostate. Bioinformatic analyses revealed that expression of *OLFM4* was significantly downregulated in advanced human prostate cancer. Our findings suggest that loss of olfactomedin 4 upregulates the hedgehog-signaling pathway and promotes progression of prostatic neoplasms.

## Results

### *Olfm4*-knockout mice sporadically develop prostatic epithelial lesions and other organ tumors

**Figure 1:**
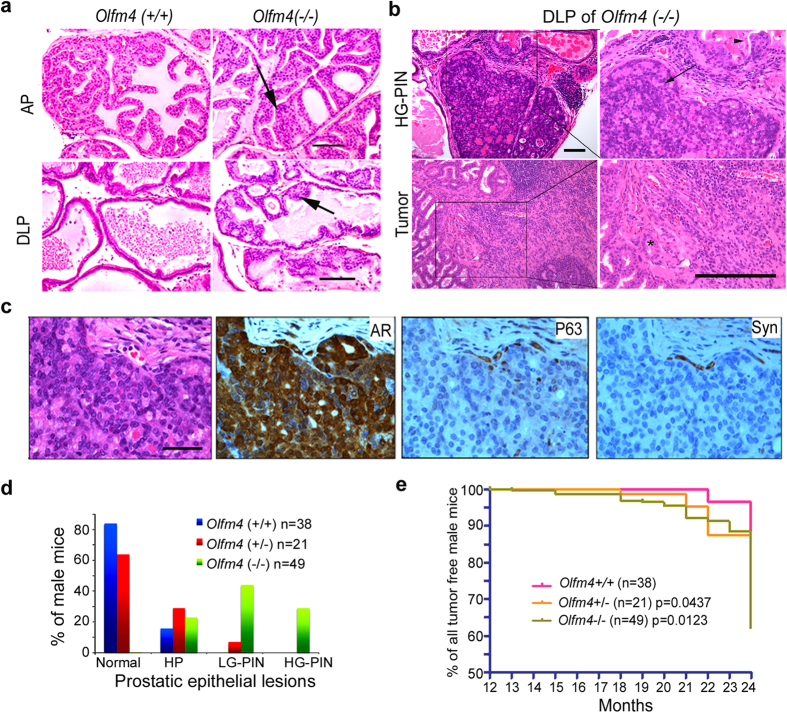
Loss of *Olfm4* leads to murine prostate neoplasia. (**a**) Representative lower-grade prostatic intraepithelial neoplasia (LG-PIN) in the anterior prostate (AP) and dorsal-lateral prostate (DLP) of HE-stained sections from littermate *Olfm4*(+/+) and *Olfm4*(−/−) mice at 18 months of age. Scale bar, 100 μm. Arrow indicates LG-PIN. (**b**) Representative prostate epithelial lesions (upper panels) and tumor (lower panels) in the DLP of HE-stained sections from *Olfm4*(−/−) mice. Arrowhead indicates hyperplasia and arrow indicates higher-grade prostatic intraepithelial neoplasia (HG-PIN) (upper right panel), and asterisk indicates microinvasion in tumor (lower right panel) at 20 months (upper panels) and 23 months (lower panels) of age. Scale bar, 100 μm. (**c**) Identification of tumor type from DLP of *Olfm4*(−/−) mice at 20 months of age using cell markers. Sections were stained with HE or with antibodies to specific cellular markers: androgen receptor (AR); the basal cell marker p63 (P63); and the neuron endocrine cell marker synaptophysin SY38 (Syn). Scale bar, 50 μm. (**d**) The percentage of prostatic epithelial lesions in 13–24-month-old *Olfm4*(+/+), (+/−), and (−/−) mice. HP, hyperplasia; LG-PIN, lower-grade prostatic intraepithelial neoplasia; HG-PIN, higher-grade prostatic intraepithelial neoplasia. (**e**) The Kaplan-Meier plot for tumor-free 13–24-month-old *Olfm4*(+/+), (+/−), and (−/−) mice. The significance of differences between experimental groups was determined by the log-rank test.

To investigate the functions of the *Olfm4* gene in murine prostate, we analyzed genomic DNA, mRNA, and protein in the prostate tissue of wild-type (*Olfm4*+/+) and *Olfm4*-knockout (*Olfm4*−/−) mice. We verified that prostate tissues from wild-type mice expressed *Olfm4* genomic DNA, Olfm4 mRNA, and Olfm4 protein, whereas those from *Olfm4*-knockout mice did not (Supplementary Fig. S1). Histopathological analysis demonstrated normal prostate tissue morphology for wild-type mice at early ages, and none developed prostatic intraepithelial neoplasia by 24 months of age. However, *Olfm4*-knockout mice sporadically developed prostatic epithelial lesions in an age-dependent manner. Prostatic epithelial hyperplasia was seen at 3–6 months of age, prostatic intraepithelial neoplasia was observed at 10–12 months of age, and higher-grade prostate intraepithelial neoplasia was observed at 18–24 months of age in *Olfm4*-knockout mice (Supplementary Table S1). Lower-grade prostatic epithelial hyperplasia (LG-PIN) was observed in the anterior prostate (AP) and dorsal-lateral prostate (DLP) of *Olfm4*-knockout mice at 18 months (Fig. 1a). Higher-grade prostatic intraepithelial neoplasia (HG-PIN), as well as inflammatory cells, was observed in the DLP of *Olfm4*-knockout mice at 20 months of age (Fig. 1b, upper panels). Microinvasion in tumor was observed in the DLP tissue of *Olfm4*-knockout mice at 23 months of age (Fig. 1b, lower panels).

Histological and immunohistochemical evaluation of DLP tissue from 20-month-old *Olfm4*-knockout mice revealed that the tumor type that developed in the *Olfm4*-knockout mice was androgen receptor (AR)-positive prostatic adenocarcinoma (Fig. 1c). The frequency of prostatic intraepithelial neoplasia lesions was increased in *Olfm4*+/− and *Olfm4*−/− mice compared with *Olfm4*+/+ mice at ages 13–24 months (Fig. 1d). In addition, we found a generally higher incidence of lung, liver, and pancreatic tumors in *Olfm4*+/− and *Olfm4*−/− mice compared with *Olfm4*+/+ mice at 13–24 months of age (Fig. 1e and Supplementary Fig. S2). These results suggest that *Olfm4* plays a pivotal tumor-suppressor role in murine prostate and other organs.

### *Olfm4* deficiency is associated with proliferation of prostate epithelial cells

**Figure 2:**
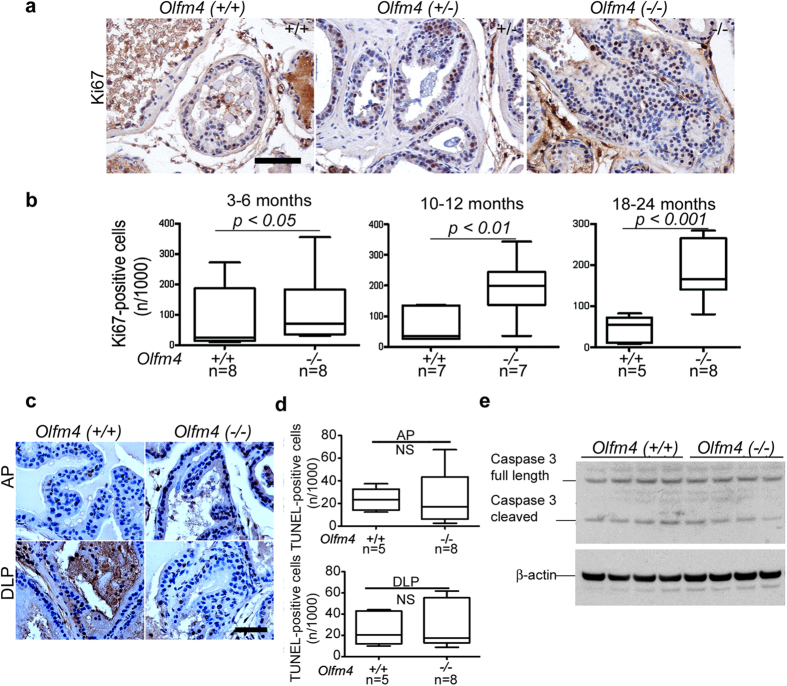
*Olfm4* deficiency enhances proliferation but does not alter apoptosis in prostate epithelial cells. (**a**) Representative images of Ki67 staining in sections of DLP tissue from *Olfm4*(+/+), *Olfm4*(+/−), and *Olfm4*(−/−) mice at 12 months of age. Bar, 100 μm. (**b**) Quantitative results of Ki67 staining in DLP of *Olfm4*(+/+) and *Olfm4*(−/−) mice at 3–6, 10–12, or 18–24 months of age. Error bars represent the SD. The significance of differences between experimental groups was determined by the Student’s t-test. (**c**) Representative images of TUNEL assays of AP and DLP from littermate 18–24-month-old *Olfm4*(+/+) and *Olfm4*(−/−) mice. Scale bar, 50 μm. Bar graphs represent the quantitative results of TUNEL staining. NS, not significant. Error bars represent the SD. The significance of differences between experimental groups was determined by the Student’s t-test. (**d**) Western-blot analysis of protein expression for caspase 3 in prostate tissues from *Olfm4*(+/+) and *Olfm4*(−/−) mice at 3 months of age. β-actin was used as a loading control.

To determine whether *Olfm4* affects proliferation or apoptosis of prostatic epithelial cells in *Olfm4*-knockout mice, we performed Ki67 immunohistochemical staining and terminal deoxynucleotidyltransferase-mediated nick end labeling (TUNEL) assays. Ki67-positive cells were detected in prostatic epithelial lesions in the DLP of 12-month-old *Olfm4*-knockout mice (Fig. 2a). The percent of Ki67-positive cells was significantly increased in the DLP of *Olfm4*-knockout mice compared with age-matched wild-type mice at different ages (Fig. 2b). However, no difference in apoptosis was observed when TUNEL assay and anti-caspase 3 Western blot results for prostate tissues of *Olfm4*-knockout mice were compared with those from wild-type mice (Fig. 2c–e). These results suggest that loss of *Olfm4* is associated with proliferation of murine prostate epithelial cells.

### *Olfm4*-knockout mouse prostate demonstrates increased levels of hedgehog signaling-pathway and target genes

**Figure 3:**
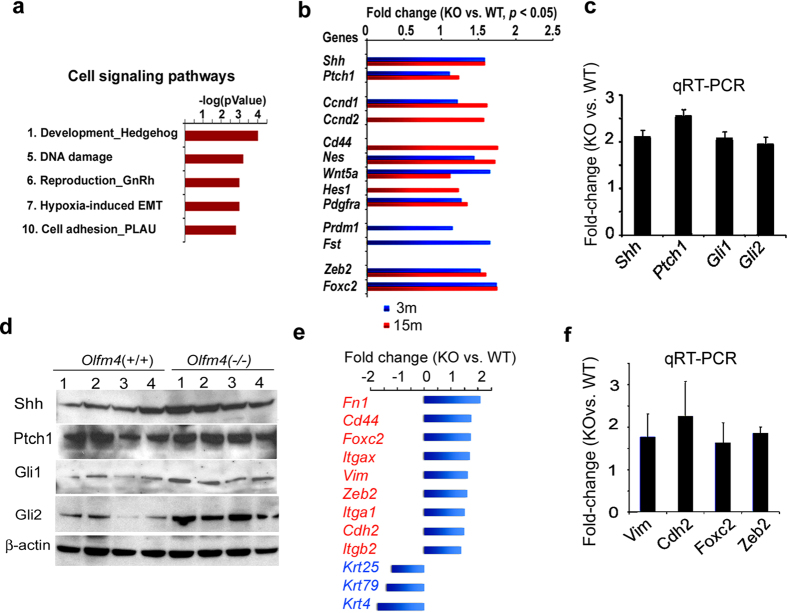
*Olfm4* deficiency is associated with expression of hedgehog signaling-pathway and target genes in murine prostate. (**a**) Cell-signaling pathways identified from GeneGo analyses of upregulated gene expression in prostate tissues of *Olfm4*(−/−) mice at 3 months of age. (**b**) Mean fold-change in expression of hedgehog signaling-pathway target genes in microarray analyses of prostate tissues from wild-type (WT; n = 4 or 3) and *Olfm4*-knockout (KO; n = 4 or 3) mice at 3 and 15 months of age. The significance of differences between experimental groups was determined by ANOVA. (**c**) Mean (±SD, n = 5) fold-change (knockout [KO] vs. wild-type [WT]) in expression of hedgehog signaling-pathway component genes in 3-month-old mouse prostate determined using qRT-PCR. The significance of differences between experimental groups was determined by the Student’s t-test. (**d**) Western-blot analysis of protein expression of hedgehog signaling-pathway components in 3-month-old *Olfm4*(+/+) and *Olfm4*(−/−) mouse prostate. β-actin was used as a loading control. (**e**) Mean fold-change in expression of upregulated (red text) and downregulated (blue text) genes for EMT, cytokeratin, and stem/progenitor-cell markers in microarray analyses of prostate tissues from *Olfm4*(−/−) mice when compared with littermate *Olfm4*(+/+) mice at 15 months of age. The significance of differences between experimental groups was determined by ANOVA. (**f**) Mean (±SD, n = 5) fold-change (knockout [KO] vs. wild-type [WT]) in expression of EMT genes in 15-month-old mouse prostate determined using qRT-PCR. The significance of differences between experimental groups was determined by the Student’s t-test.

Gene-microarray data analysis indicated that the hedgehog-signaling pathway was significantly changed in *Olfm4*-knockout mouse prostate (Fig. 3a). The mRNA expression of the hedgehog signaling-pathway genes *Shh* and *Ptch1*, *Shh*’s direct-target genes *Ccnd1*, *Ccnd2*, *Foxc2*, and *Prdm1*, and *Shh*’s indirect-target genes, including the stem-cell signaling network and stem-cell markers *Cd44*, *Nes*, *Wnt5a*, *Hes1*, *Pdgfra*, and *Fst*, were significantly upregulated in prostate tissues from both or either 3-month-old and 15-month-old *Olfm4*-knockout mice compared with those from wild-type mice (Fig. 3b). Using qRT-PCR, we further found an approximately 2-fold or higher increase in the expression of *Shh*, *Ptch1*, *Gli1*, and *Gli2* in prostate tissues from 3-month-old *Olfm4*-knockout mouse compared with those from wild-type mice (Fig. 3c). The increased expression of hedgehog signaling-pathway component genes in the prostate of *Olfm4*-knockout mice compared with wild-type mouse prostate was also verified at the protein level (Fig. 3d). Interestingly, we found that some hedgehog-signaling target genes (including the prostate stem/progenitor cell marker gene *Cd44*, as well as some epithelial-to-mesenchymal [EMT] marker genes, including *Zeb2*, *Foxc2*, *Vim*, *Fn1*, and *Cdh2*) were significantly upregulated in prostate tissues from 15-month-old *Olfm4*-knockout mice compared with those from wild-type mice (Fig. 3e,f). In contrast, the cytokeratin genes *Krt25*, *Krt4*, and *Krt79* were found to be significantly downregulated in prostate tissues from 15-month-old *Olfm4*-knockout mice compared with those from wild-type mice (Fig. 3e). These results suggest that loss of *Olfm4* leads to enhanced hedgehog signaling-pathway and pathway-target gene expression.

### *OLFM4* downregulates hedgehog signaling-pathway components in human prostate-cancer cell lines

**Figure 4:**
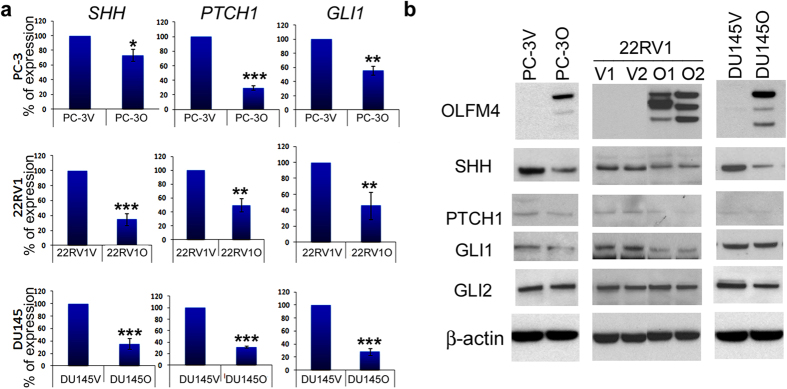
*OLFM4* downregulates hedgehog signaling-pathway components in human metastatic prostate-cancer cells. The *OLFM4* stably expressing human metastatic prostate-cancer cell clones PC-3V (vector-GFP tag), PC-3O (*OLFM4*-GFP tag); DU145V (vector-GFP tag), DU145O (*OLFM4*-GFP tag); 22RV1V (vector-GFP tag clone 1 and 2), and 22RV1O (*OLFM4*-GFP tag clone 1and 2; clone 1 data are presented in panel a) were established. (**a**) qRT-PCR analysis of *SHH*, *PTCH1*, and *GLI1* in prostate-cancer cell clones. Data represent the mean (±SD) percent expression in *OLFM4*-GFP tag-expressing cell clones compared with vector-GFP tag-expressing cell clones (value set at 100%) (n = 3). *P < 0.05; **P < 0.01; ***P < 0.001. The significance of differences between experimental groups was determined by the Student’s t-test. (**b**) Western-blot analysis of protein expression for OLFM4, SHH, PTCH1, GLI1, and GLI2 in prostate-cancer cell clones. β-actin was used as a loading control.

We have previously verified that human metastatic prostate-cancer cells lack *OLFM4* expression^12^. We next studied hedgehog signaling-pathway components in human prostate-cancer cell lines stably expressing the *OLFM4* gene. We found that expression of the hedgehog-signaling components *SHH*, *PTCH1*, and *GLI1* was reduced between 30–70% in *OLFM4*-expressing prostate-cancer cells compared with control vector-transfected cells (Fig. 4a). Protein expression of the hedgehog signaling-pathway components SHH, PTCH1, GLI1, and GLI2 was also generally reduced in *OLFM4*-expressing prostate-cancer cells compared with control vector-transfected cells (Fig. 4b). In addition, AR-positive, androgen-sensitive VCaP prostate-cancer cells transiently overexpressing *OLFM4* displayed reduced expression of *SHH* and *GLI* at the mRNA level but not the protein level (Supplementary Fig. S3). The expression of *PTCH1* was not changed may be due to the specific cellular context (Supplementary Fig. S3). Collectively, these results suggest that the expression of OLFM4 in human prostate-cancer cell lines that normally lack OLFM4 expression inhibits the hedgehog-signaling pathway.

### *OLFM4* protein directly interacts with sonic hedgehog (SHH) protein, reduces level of SHH protein in the culture media of PC-3 cells, and inhibits GLI-reporter activity

**Figure 5:**
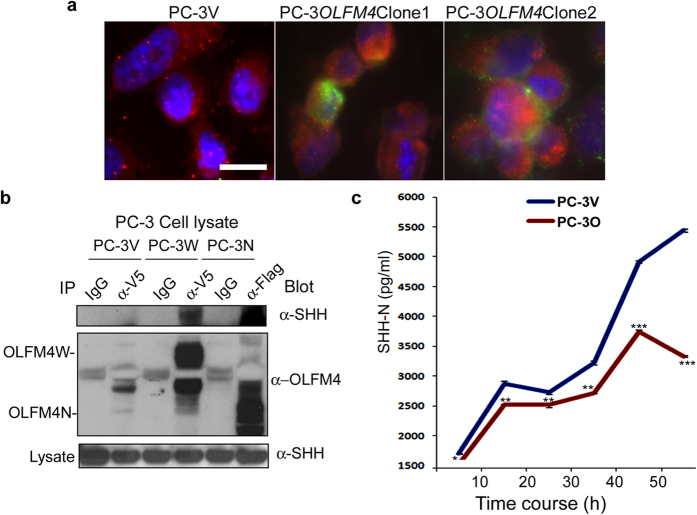
OLFM4 protein directly interacts with SHH protein and reduces SHH protein level in the culture media of PC-3 cells. (**a**) Immunofluorescent staining of PC-3 cells transfected with *OLFM4*-V5 tag (PC-3*OLFM4* clones) or vector control (PC-3V), using anti-V5 (green) anti-SHH (red) antibodies. Nuclei were counterstained with DAPI (blue). Scale bar, 50 μm. (**b**) Coimmunoprecipitation analysis of OLFM4 and SHH. Cell lysates of PC-3 vector control-transfected cell clones (PC-3V), PC-3 cell clones stably expressing *OLFM4*-V5 tag (PC-3W), or PC-3 cell clones expressing *OLFM4*-N (a truncated deletion of *OLFM4*)-Flag tag (PC-3N) were immunoprecipitated with anti-V5 or anti-Flag (or normal IgG) antibody. Immunoprecipitates were subjected to Western-blot analysis with anti-SHH (upper panel) or anti-OLFM4 (middle panel) antibody. Total lysate subjected to Western-blot analysis with anti-SHH antibody was used as a loading control (lower panel). IgG indicates a normal IgG used as a negative control in the immunoprecipitation assays presented. (**c**) Time course of SHH protein secretion into the culture media of vector control-transfected PC-3 (PC-3V) and *OLFM4*-transfected PC-3 (PC-3O) cell clones. The cell-culture media (RPMI 1640 containing 0.5% FBS) was harvested from 3 individual wells of 12-well plates after culturing for 6, 18, 24, 30, 42, or 54 h. SHH secretion was determined by ELISA. Data represent the mean ± SD (n = 3). *P < 0.05, **P < 0.01, ***P < 0.001. The significance of differences between experimental groups was determined by ANOVA.

To investigate whether OLFM4 protein directly or indirectly affects the hedgehog-signaling pathway, we examined OLFM4 and SHH localization in *OLFM4*-expressing PC-3 cells. The OLFM4 and SHH proteins were observed to be colocalized in these cells (Fig. 5a). The physical interaction between OLFM4 and SHH was also detected in PC-3 cells stably expressing *OLFM4* or *OLFM4*-N (a truncated deletion of *OLFM4*) using coimmunoprecipitation analysis (Fig. 5b). SHH protein secretion was significantly reduced in the conditioned culture media of PC-3 *OLFM4*-expressing cell clones compared with PC-3 vector control-transfected cell clones (Fig. 5c). These results suggest that OLFM4 downregulated both cellular and secreted levels of SHH protein.

**Figure 6:**
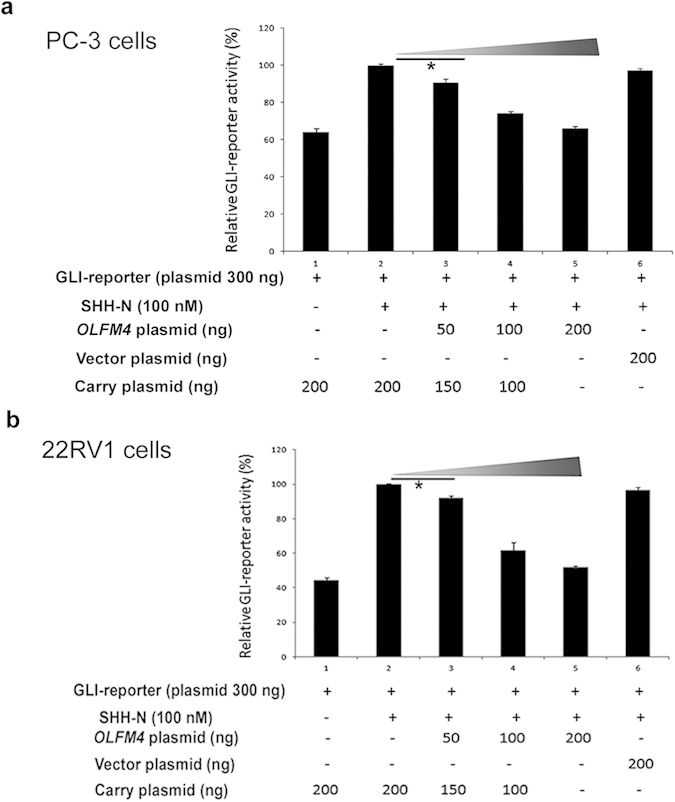
OLFM4 inhibits GLI-reporter activity in prostate-cancer cells. Effects of the *OLFM4* gene on GLI-reporter activity in PC-3 cells (**a**) and 22RV1 cells (**b**). Bar graph represents the relative GLI-reporter activity that was normalized by using cotransfection with *Renilla* luciferase and detected using the dual-luciferase reporter assay system. The mean percent was obtained by comparing activity in triplicate transfections for each experimental condition to the activity for the SHH-N–treated sample (number 2; value set at 100%). SHH-N protein (100 nM) was added 48 h after transfection, and GLI-reporter activity was measured 24 h later. Carry plasmid indicates plasmid carried empty vector. Data represent the mean ± SD of triplicate experiments. *P < 0.05. The significance of differences between experimental groups was determined by ANOVA. Shadow triangle indicates dose of *OLFM4* cDNA plasmid.

To study whether OLFM4 protein directly inhibits hedgehog signaling-pathway activity, we performed GLI-reporter activity assays with human prostate-cancer cells. We found dose-dependent inhibitory effects when *OLFM4* plasmid was cotransfected with GLI-reporter plasmid in PC-3 (Fig. 6a) and 22RV1 cells (Fig. 6b), as well as in 293T cells (Supplementary Fig. S4a). Exogenous OLFM4 protein significantly inhibited GLI-reporter activity in 293T cells (Supplementary Fig. S4b). Further, OLFM4 protein and cyclopamine synergistically inhibited GLI-reporter activity in 293T cells (Supplementary Fig. S4b). These findings suggest that OLFM4 protein is a natural antagonist of the hedgehog-signaling pathway.

### Expression of *OLFM4*, *SHH*, and hedgehog signaling-pathway target genes in human prostate-cancer specimens reflects expression patterns seen in prostate tissue from *Olfm4*-knockout mice

**Figure 7:**
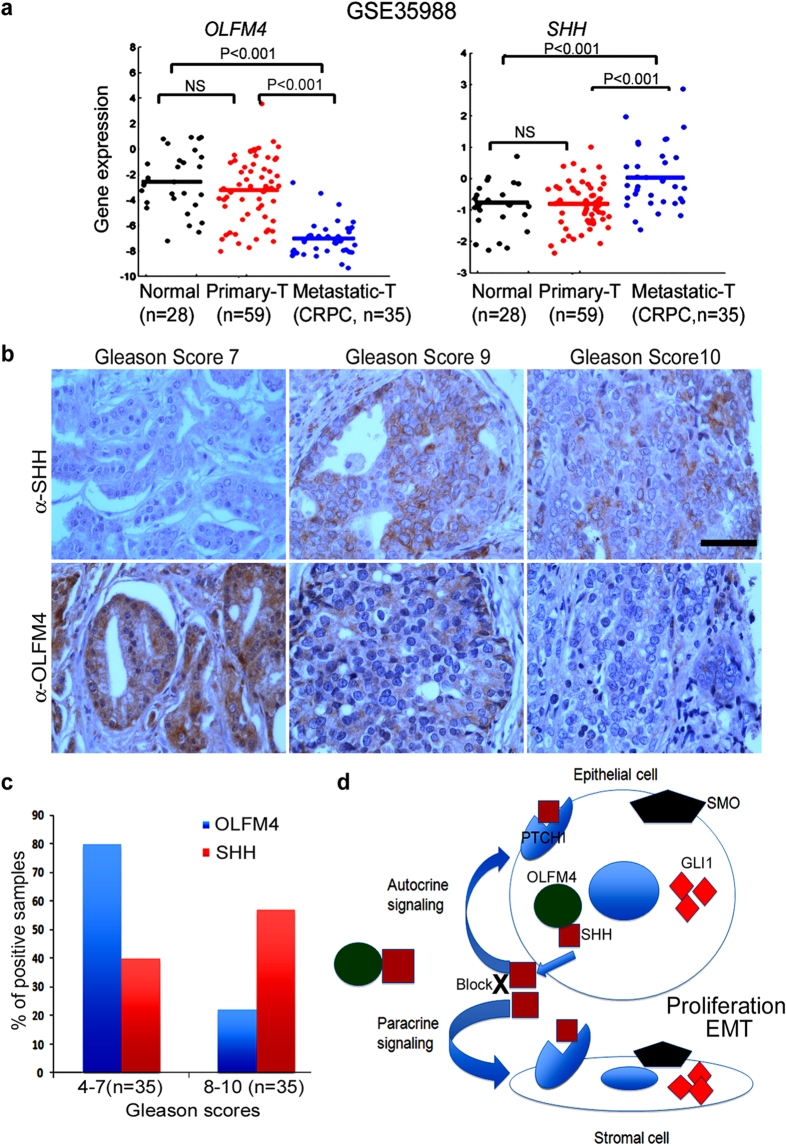
*OLFM4* expression is decreased and *SHH* expression is increased in human prostate-cancer progression. (**a**) Gene-expression levels in published human prostate tissue GSE35988 microarray data. Scott plot graphs represent the relative expression of *OLFM4* and *SHH* in normal prostate, primary prostate tumors, and metastatic prostate tumors. NS, not significant. CRPC, castrate-resistant prostate cancer. The significance of differences between any 2 stages was determined by Mann-Whitney U tests. (**b)** Representative images of immunohistochemistry analysis of SHH and OLFM4 expression in human prostate-cancer tissue-array specimens with different Gleason scores. Scale bar, 100 μm. (**c**) Bar graph represents quantitation of immunohistochemistry staining results from (**b**). (**d**) A model illustrating the function of OLFM4 in regulating hedgehog signaling-pathway activities. OLFM4 protein binds to the SHH protein and blocks its binding to the PTCH1 receptor, therefore inhibiting autocrine and paracrine signaling-pathway activities that regulate cellular proliferation and EMT.

Given our results observed in *Olfm4*-knockout mice and human prostate-cancer cells, we sought to analyze the expression of *OLFM4*, *SHH*, and hedgehog signaling-pathway target genes in the GSE35988^19^ and GDS2545 (GEO profiles) datasets. *OLFM4* was significantly downregulated in metastatic tumor tissue when compared with normal prostate tissue, whereas *SHH* was significantly upregulated in metastatic tumor tissue when compared with normal prostate tissue (Fig. 7a and Supplementary Fig. S5, upper row). The hedgehog-signaling direct-target genes *CCND1*, a cell-cycle regulator gene^20^,^21^, and *PDGFRA*^22^ were upregulated in human primary and metastatic tumors when compared with normal prostate tissue (Supplementary Fig. S5, lower row). An indirect regulator gene, *WNT5A*^23^, was upregulated in metastatic tumors when compared with normal prostate tissue (Supplementary Fig. S5, lower row). *WNT5A* mediates the protein kinase C pathway to promote invasion and metastasis^24^, while the *PDGFRA* gene (which encodes platelet-derived growth factor receptor alpha) upregulates the ERK signaling pathway in basal cell carcinoma^22^.

The expression of OLFM4 and SHH proteins was examined in prostate-cancer tissue-array samples using immunohistochemistry. We detected positive OLFM4 staining, but not SHH staining, in Gleason score 4–7 prostate-cancer specimens, while OLFM4 staining was reduced or lost and SHH staining was increased in Gleason scores 8–10 prostate-cancer specimens (Fig. 7b and Supplementary Table S2). The percentage of OLFM4- and SHH-positive prostate-cancer specimens was 80% and 40% in Gleason scores 4–7, and 22% and 57% in Gleason scores 8–10, respectively (Fig. 7c). Loss of protein expression of OLFM4 and increased expression of SHH were significantly associated with high grade of prostate cancer (Supplementary Table S2). These results suggest that reduced or lost *OLFM4* gene expression and increased *SHH* gene expression are associated with human prostate neoplastic progression.

## Discussion

*OLFM4* is located on chromosome 13q14.3^11^, which is frequently deleted in many human cancers, including prostate cancer. We have found previously that deletion of the *OLFM4* gene is associated with the progression of human prostate cancer^16^. Further, our functional studies of *OLFM4* in human prostate-cancer cells have demonstrated that restoration of *OLFM4* expression in the metastatic prostate-cancer cell line PC-3 significantly inhibited cancer-cell growth, invasion, and metastasis^12^. Our previous findings have strongly suggested that *OLFM4* plays a critical role in regulating progression of human prostate cancer.

*Olfm4*-knockout mice have previously been shown to demonstrate normal early development, and *Olfm4* does not appear to be essential for normal development and growth in mice^25^. Because *Olfm4* is normally expressed in the mouse prostate, we used the *Olfm4*-knockout mouse model to examine the relationship between *Olfm4* deficiency and prostate neoplastic progression. In this study, we found that approximately 70% of *Olfm4*-knockout mice developed prostatic epithelial lesions. The frequency of tumor formation in the liver, lung, and pancreas was also significantly increased in older *Olfm4*-knockout mice. Thus, the *Olfm4* gene seems to serve as a tumor suppressor in the progression of prostate cancer and other tumors.

Gene-expression profile studies of *Olfm4*-knockout mouse prostate tissue at 3 and 15 months of age demonstrated that loss of *Olfm4* significantly altered gene-expression levels of hedgehog signaling-pathway genes and target genes. Given this gene-expression profile, we explored a potential novel function of Olfm4—that of modulating the hedgehog-signaling pathway.

Overexpression of hedgehog has been reported to initiate prostate stem/progenitor cell transformation in normal murine prostate tissue and to cause lesions with characteristic prostatic intraepithelial neoplasia or/and prostatic cancer in murine prostate^10^. Furthermore, activation of the hedgehog-signaling pathway by using stable transfection of GLI in rodent prostate-cancer lines has demonstrated that hedgehog-pathway activity upregulates EMT and determines metastatic potential^4^. Interestingly, we found that *Olfm4*-knockout murine prostate displayed neoplastic progression and elevated expression of hedgehog signaling-pathway component and target genes, as well as EMT marker genes. We also obtained more evidence of OLFM4’s ability to inhibit hedgehog-signaling activity in human prostate-cancer cell lines. It has been previously shown that overexpression of hedgehog ligands in human prostate-cancer cell lines promotes growth, while a hedgehog signaling-specific inhibitor significantly reduces growth in a human prostate-cancer cell xenograft model^4^. In this study, we observed that restoration of *OLFM4* in human metastatic prostate-cancer cell lines lacking expression of *OLFM4* inhibited hedgehog-signaling activity. Based on our new findings, we postulate that OLFM4 protein binds to the SHH protein, blocks the binding of SHH to its receptor, PTCH1, and therefore inhibits hedgehog signaling-pathway activities (Fig. 7d). Conversely, loss of OLFM4 protein would increase hedgehog signaling-pathway activity and cross-talk with other pathways that mediate hedgehog-signaling pathway activities.

Taken together, our findings suggest that OLFM4 plays a critical role in regulating progression of prostate cancer and might be useful as a novel candidate biomarker for improving diagnostic/prognostic accuracy and as a likely target for therapeutic approaches to prostate cancer.

## Methods

### Mouse procedures

All animal experiments were approved by the Animal Care and Use Committee of the National Heart, Lung, and Blood Institute (NHLBI). Animal care was performed in accordance with relevant institutional and national guidelines and regulations in the animal facilities of the National Institutes of Health (NIH). Generation of *Olfm4*-knockout mice has been described previously^25^. The *Olfm4*-knockout mice were maintained by crossing *Olfm4*(+/−) mice. The animals were genotyped using PCR with primers as described previously^25^. The genetic background of these mice was 50% 129/SV and 50% National Institutes of Health Black Swiss.

### Mouse tissue collection, histopathological analysis, and immunohistochemistry

Prostate microdissection was performed following a previously described procedure^26^; other tissues were also harvested at the time of sacrifice. Histological and immunohistochemical analyses were performed on formalin-fixed paraffin sections as previously described^16^. Histological analysis was performed on HE-stained tissue slides on a blinded basis by Dr. Victoria Hoffmann, DVM, DACVP (Division of Veterinary Resources/NIH), Dr. Jaime Rodriguez-Canales MD (a pathologist in the Laboratory of Pathology, National Cancer Institute, NIH), and one of the authors (Dr. H. Li, MD, PhD). LG-PIN (I–II) and HG-PIN (III–IV) were identified according to recommendations for scoring mouse prostate-cancer models that have been published previously^26^,^27^. Immunohistochemical staining was performed with the following antibodies: anti-AR (PG-21; Millipore); anti-p63 (Santa Cruz Biotechnology, Inc.); anti-synaptophysin SY38 (Abcam); and anti-Ki67 antigen (polyclonal, NovoCastra Laboratories). Secondary antibodies, Super Sensitive MultiLink, and Super Sensitive Label were purchased from BioGenex. Dark brown color was developed with chromagen (BioGenex) and counterstained with hematoxylin. The Ki67-staining samples were evaluated by counting a total of thousands cells in 5 areas of each sample.

All images were acquired using an Olympus BX51 microscope (Olympus) and Qimaging Camera with Q Capture pro software (Qimaging). Images were acquired using the ×60 Uplan Apo objective (1.42 oil), then imported into Adobe Photoshop for presentation.

### Immunofluorescent staining

Immunofluorescent staining of human prostate-cancer cells was performed with anti-V5 (Invitrogen) or anti-SHH (Millipore) antibodies, and immunofluorescent staining of mouse prostate tissues was performed with an anti-OLFM4 polyclonal antibody^28^, followed by secondary antibody (Alexa Fluor 488-conjugated goat anti-rabbit or anti-mouse or Alexa Fluor 596-conjugated goat anti-rabbit or anti-mouse; all from Invitrogen). Nuclei were counterstained with DAPI (4′,6-diamidino-2-phenylindole dihydrochloride).

All images were acquired using an Olympus BX51 microscope and Qimaging Camera with Q Capture pro software. Images were acquired using the ×60 Uplan Apo objective (1.42 oil), then imported into Adobe Photoshop for presentation.

### Gene-microarray analysis

Total RNA was purified from whole prostate tissue from wild-type and *Olfm4*-knockout mice at 3 and 15 months of age using the RNeasy Plus Mini Kit (Qiagen). cDNA microarray analyses were performed by the National Institute of Diabetes and Digestive and Kidney Diseases (NIDDK) Core Facility at the NIH using Affymetrix Mouse Genome 430 2.0 Array GeneChips (Affymetrix). Five biological replications were used for the wild-type or *Olfm4-*knockout prostate RNA extracted from 5 individual mice. The microarray signals were analyzed using the Affymetrix RMA algorithm. Analysis of variance results, false discovery rate reports, and heatmaps were generated using Partek Genomic software 6.5 (Partek). Enrichment analyses of GO categories, which include disease markers, as well as biological process and pathway analyses, were performed using MetaCore web-access software (http://www.genego.com).

### Genomic DNA PCR and qRT-PCR analysis

qRT-PCR was conducted as previously described^12^. The TaqMan real-time PCR primers and probes were purchased from Applied Biosystems. Relative expression was calculated using ΔΔCt methods. Genomic DNA and total RNA were extracted from mouse prostate and genomic DNA PCR^25^ and semi-quantitative RT-PCR^12^ were performed as previously described.

### Western-blot analysis

Samples were separated using 4–12% polyacrylamide gel electrophoresis, transferred to polyvinyldiene fluoride membrane, and hybridized with anti-OLFM4 (Sino Biological Inc.), anti-SHH, PTCH1, GLI1, or GLI2 or anti-Shh, Ptch1, Gli1, or Gli2 (Abcam), anti-β-actin (Santa Cruz Biotechnology, Inc.), or anti-caspase 3 (Cell Signaling Technology, Inc.) antibodies overnight at 4 °C. The membranes were then incubated with secondary antibody and signal developed with Amersham ECL Western-blotting detection reagents (GE Healthcare).

### Generation of stably expressing *OLFM4*-GFP prostate-cancer cell clones

PC-3, 22RV1, and DU145 human prostate-cancer cell lines were purchased from ATCC. We have previously verified that human metastatic prostate-cancer cells lack *OLFM4* expression^12^. The construction of vector-V5 tag, *OLFM4*-V5 tag, and truncated mutant *OLFM4*-Flag tag plasmids and their stable expression in plasmid cell clones have been previously described^12^,^16^. PC-3V (vector-transfected control) clone, PC-3W (full-length *OLFM4*-V5 tag-expressing) clone, and PC-3N (expressing a truncated mutant of *OLFM4*-Flag tag in which the olfactomedin domain is missing) clone cells were cultured in RPMI 1640 media containing 10% fetal bovine serum (FBS) until 90% confluent^16^,^28^. The pCMV-6-AC-GFP tag-vector and pCMV-6-*OLFM4*-GFP tag plasmids were purchased from Origene. Stably expressing *OLFM4*-green fluorescent protein (GFP) tag-expressing prostate-cancer cell clones (O) or vector-GFP tag-transfected control prostate-cancer cell clones (V) were established following previously described protocols^12^,^16^.

### Generation of transiently overexpressing *OLFM4*-GFP prostate-cancer cells

The human AR-positive, androgen-sensitive prostate-cancer cell line VCaP was purchased from ATCC. VCaP cells were maintained in DMEM containing 10% FBS. For transient transfection, the cells (5 × 10^5^) were plated in 6-well plates and transfection performed with pCMV-6-AC-GFP tag-vector or pCMV-6-*OLFM4*-GFP tag plasmids, using Lipofectamine 2000 transfection reagent (Invitrogen) according to the manufacturer’s instructions.

### Coimmunoprecipitation assays

For coimmunoprecipitation assays, PC-3 cells were harvested and lysed in immunoprecipitation lysis buffer^29^. Culture media were harvested from 48-h cell cultures in T75 flasks. Cell lysates (500 μg in 0.5 mL) were mixed with 2 μg of normal IgG or antibody to Flag (Sigma-Aldrich) or V5 (Invitrogen), then incubated for 3 h at 4 °C. Following this incubation, 100 μL of rec-Protein G-Sepharose 4B conjugate (Invitrogen) was added and incubated with mixing overnight at 4 °C. After centrifugation at 3,000 × *g* for 1 min, supernatants were aspirated and discarded. Pellets were washed in lysis buffer 3 times for 15 min. Sample buffer (30 μL) was added to the agarose pellets, which were then boiled for 10 min. Samples were then subjected to Western-blot analysis.

### GLI-reporter assays

The Cignal reporter and Cignal lenti reporter assay kits were purchased from Qiagen. Transient transfection of 293T cells (ATCC) or prostate-cancer cells with Cignal lentiviral particles or GLI-reporter plasmids was performed following the manufacturer’s instructions. Briefly, for Cignal lenti reporter transfection, 1 × 10^4^ cells/well of 293T cells were plated onto 96-well plates and transfected with Cignal lentiviral particles following the manufacturer’s instructions. After 48 h, the cells were treated with different concentrations of OLFM4 protein (Sino Biological Inc.) (1.5–6.0 μM) and/or SHH-N (R&D Systems) (200 nM) in Opti-MEM containing 0.5% FBS for 24 h in 96-well plates. In some assays, cyclopamine (1 μM) was included in the culture media. For transient cotransfection of GLI-reporter plasmid and vector control plasmid, *OLFM4* cDNA plasmid, and/or carry plasmids (empty vector only), 2 × 10^6^ cells/well of 293T cells were plated onto 6-well plates or 1 × 10^5^ cells/well of PC-3 or 22RV1 cells were plated onto 24-well plates. The cells were transfected with GLI-reporter plasmids combined with *OLFM4* cDNA plasmids or control vector plasmids and/or carry plasmids. After 48 h, the cells were treated with 100 nM SHH-N in Opti-MEM containing 0.5% FBS for 24 h in 96-well plates. GLI-reporter activity was detected using a dual-luciferase reporter assay system (Promega).

### SHH ELISA assays

SHH ELISA was performed using the ab100639-Sonic Hedgehog Human ELISA Kit (Abcam). Briefly, cell-culture media samples were harvested from 3 individual wells of 12-well plates after culturing for 6, 18, 24, 30, 42, or 54 h and diluted 10-fold by using 1× assay diluent B, then incubated overnight at 4 °C in the ELISA plate. The OD values were measured at 450 nm with a 1420 Multilabel Counter (Perkin Elmer Inc.).

### TUNEL assays

TUNEL assays were performed according to the instructions for the *In Situ* Cell Death Detection Kit (Roche). The proportion of positive cells was determined by counting a total of thousands cells in 5 areas of each sample.

#### Human prostate cancer tissue array and immunohistochemistry

Prostate cancer tissue arrays were purchased from US Biomax (catalogue number PR803). The immunohistochemical staining with OLFM4 antibody and SHH antibody (ab53281, abcam) and quantitation of immunohistochemistry staining results was performed as previously described^16^.

### Statistical analysis

Statistical analyses were performed using the Student’s t-test, ANOVA, log-rank test, or the Mann-Whitney U test. *P*-values <0.05 were considered statistically significant.

### Accession website

The GEO submission number for the microarray data discussed in this publication is GSE39989. The GEO database is available at http://www.ncbi.nlm.nih.gov/geo.

## Additional Information

**How to cite this article**: Li, H. *et al.* Olfactomedin 4 deficiency promotes prostate neoplastic progression and is associated with upregulation of the hedgehog-signaling pathway. *Sci. Rep.* **5**, 16974; doi: 10.1038/srep16974 (2015).

## Supplementary Material

Supplementary Information
