# Supplementary material for: Olfactomedin 4 deficiency promotes prostate neoplastic progression and is associated with upregulation of the hedgehog-signaling pathway
Source: Sci Rep. 2015 Nov 19;5:16974. doi: 10.1038/srep16974 (PMC4652203; doi:10.1038/srep16974)
Supplement: Supplementary Information [file srep16974-s1.doc]

**Olfactomedin 4 deficiency promotes prostate neoplastic progression and is associated with upregulation of the hedgehog-signaling pathway**

Hongzhen Li, Wenli Liu, Weiping Chen, Jianqiong Zhu, Chu-Xia Deng, and Griffin P. Rodgers


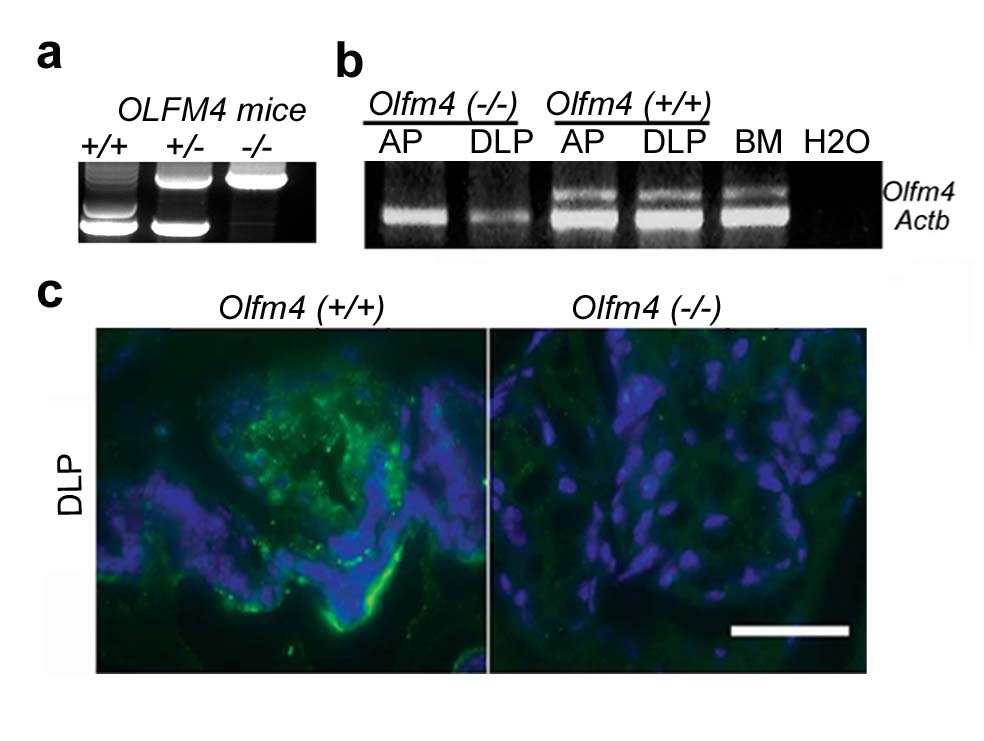


**Supplementary Figure S1. Validation of *Olfm4* gene deletion in prostate tissues from *Olfm-* knockout mice.** (**a**) PCR genotype of *Olfm4*(+/+), (+/-), and (-/-) mice at one month of age. (**b**) Semi-quantitative RT-PCR for detecting *Olfm4* mRNA expression in anterior prostate (AP) and dorsal-lateral prostate (DLP) mouse tissues at three months of age. BM is mouse bone marrow mRNA as a positive control. H2O is water as a negative control. *Actb* was used as an internal control. (**c**) Representative images of fluorescent immunostaining of frozen DLP tissue with OLFM4 antibody from *Olfm4*(+/+) and *Olfm4*(-/-) mice at three months of age. Nuclei were counterstained with DAPI (blue). Scale bar, 50 μm.


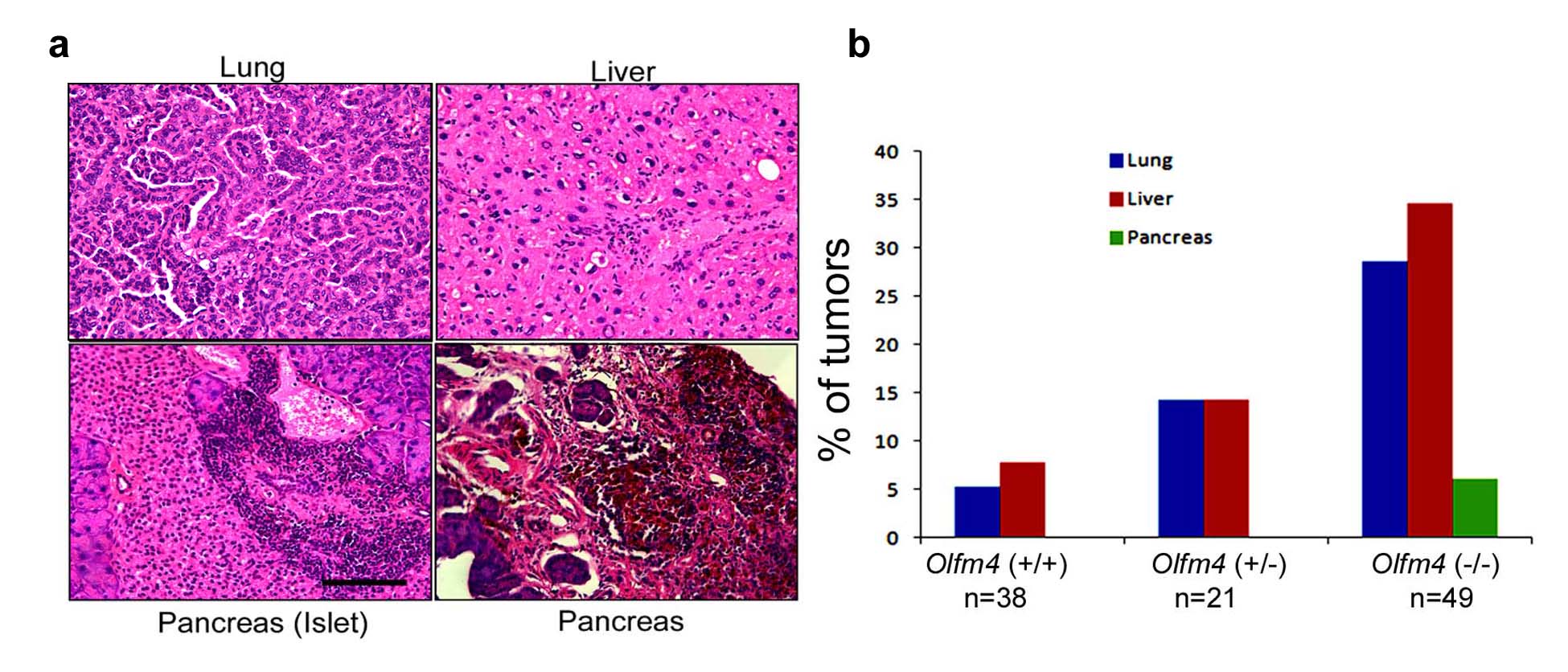


**Supplementary Figure S2. *Olfm4* deficiency increased tumor incidence in the lung, liver, and pancreas.** (**a**) HE staining of lung, liver, and pancreas tumors. The tumors were harvested from *Olfm4*(-/-) mice at 13–24 months of age. Scale bar, 50 μm. (**b**) The percentage of *Olfm4*(+/+), (+/-), and (-/-) mice at 13–24 months of age with lung, liver, or pancreas tumors.


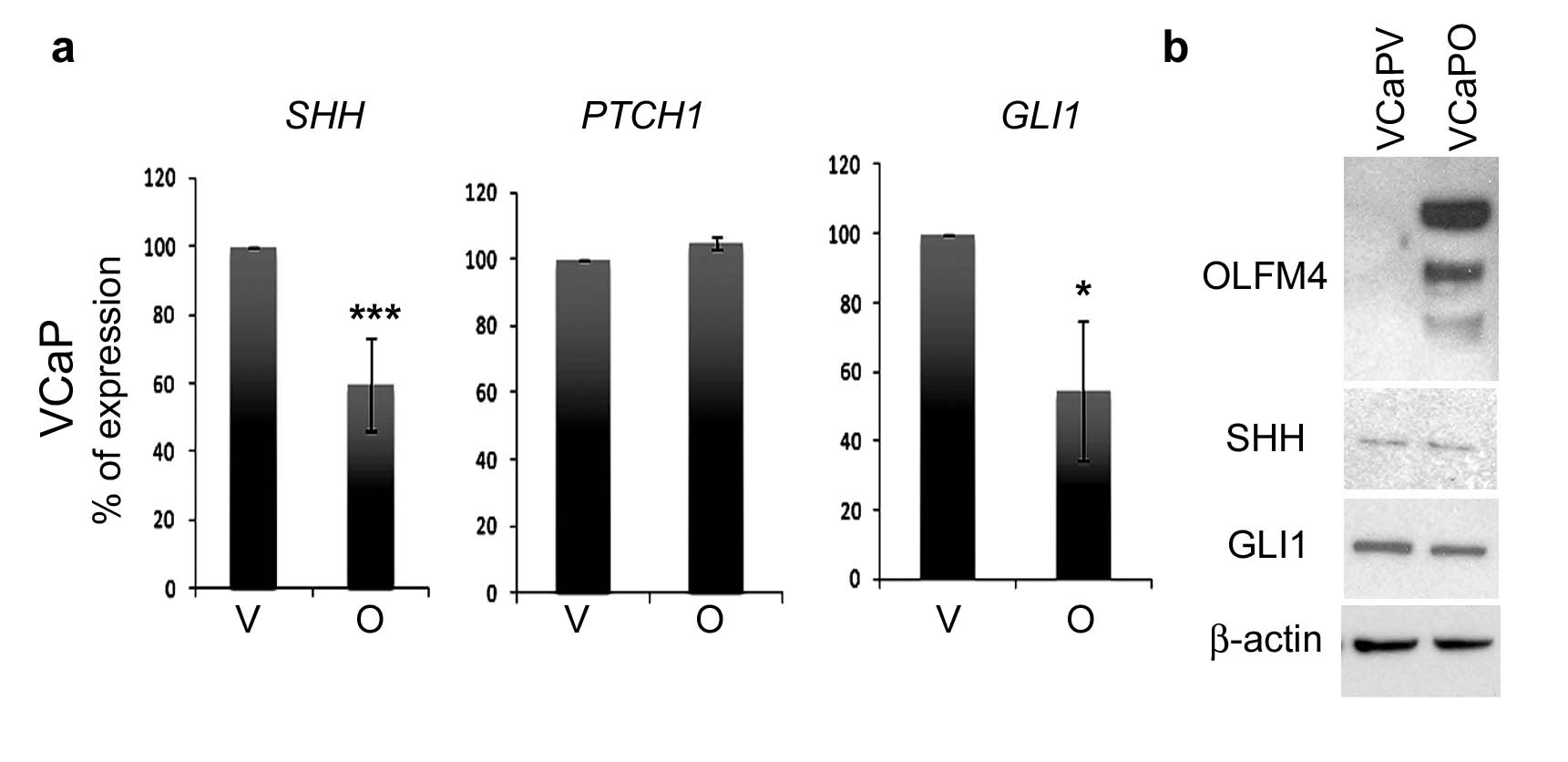


**Supplementary Figure S3. *OLFM4* downregulates mRNA expression of *SHH* and *GLI1* after 48h transient-transfection in human AR-positive, androgen-sensitive VCaP prostate-cancer cells.** Human AR-positive, androgen-sensitive VCaP prostate-cancer cells were transiently transfected with the *OLFM4* gene. (**a)** qRT-PCR analysis of *SHH*, *PTCH1*, and *GLI1* in VCaP cells after transient transfection for 48h. Non-transfected (-); transfected with vector-GFP tag (V); and transfected with *OLFM4*-GFP tag (O). Data represent the mean (±SD) percent expression in *OLFM4*-GFP tag-expressing cells compared with vector-GFP tag-expressing cells (value set at 100%) (n=5). **P* < 0.05; ***P* < 0.01. The significance of differences between experimental groups was determined by the Student’s t-test. (**b**) Western-blot analysis of protein expression for OLFM4, SHH, and GLI1 in VCaP cells. β-actin was used as a loading control.


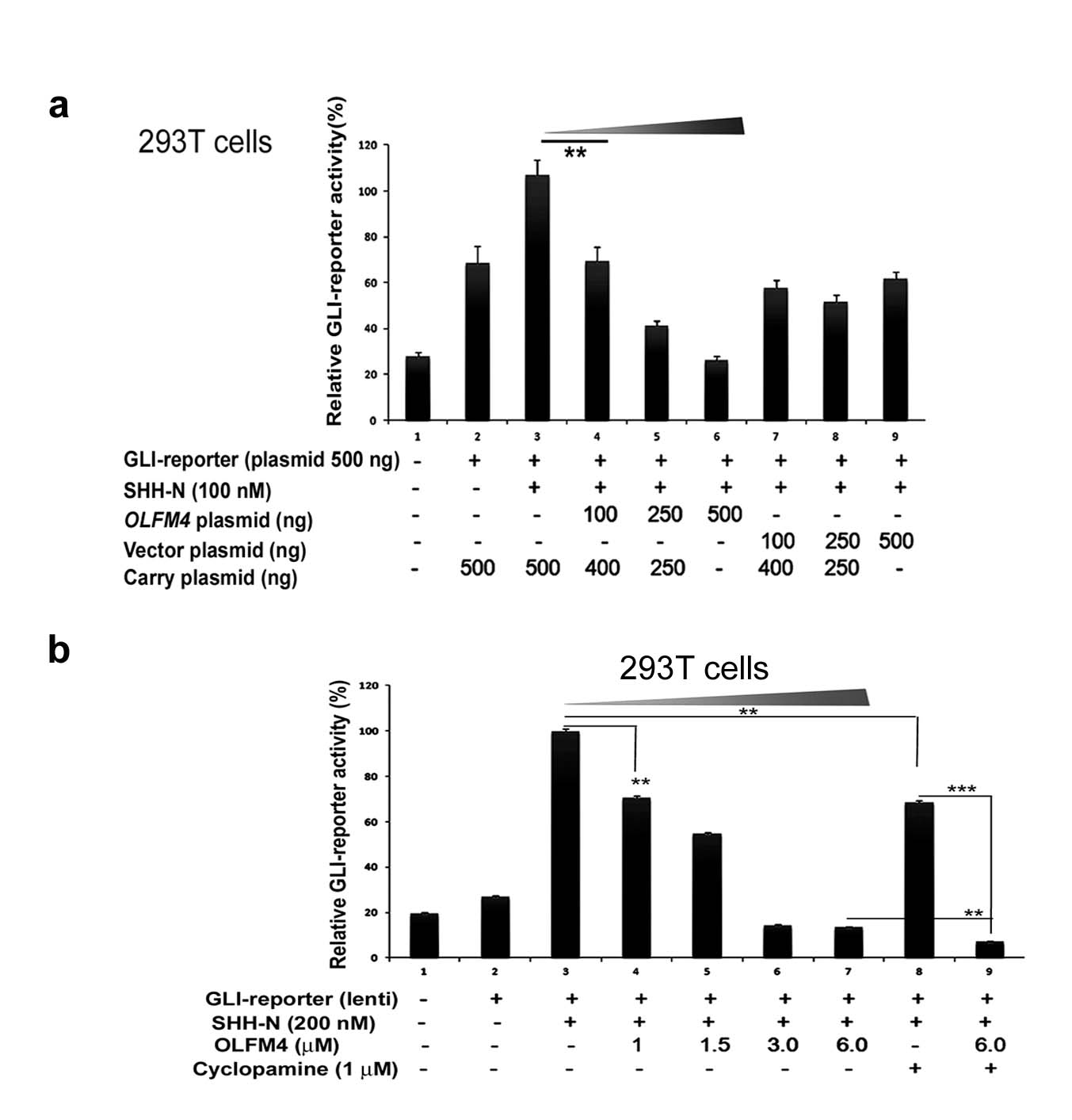


**Supplementary Figure S4. OLFM4 inhibits GLI-reporter activities in 293T cells.** (**a**) Effects of the *OLFM4* gene on GLI-reporter activity in 293T cells. Bar graph represents the relative GLI-reporter activity that was normalized by using cotransfection with *Renilla* luciferase and detected using the dual-luciferase reporter assay system. The mean percent was obtained by comparing activity in triplicate transfections for each experimental condition to the activity for the SHH-N–treated sample (number 3; value set at 100%). SHH-N protein (100 nM) was added 48 h after transfection, and GLI-reporter activity was measured 24 h later. Carry plasmid indicates plasmid carried empty vector. Data represent the mean ± SD of triplicate experiments. ***P* < 0.01. Shadow triangle indicates dose of *OLFM4* cDNA plasmid. (**b**) Effects of OLFM4 protein on GLI-reporter activity in 293T cells. Bar graph represents the relative GLI-reporter activity that was normalized by using cotransfection with *Renilla* luciferase and detected using the dual-luciferase reporter assay system. Cignal lenti reporter transfection was performed with 1×10^4^ cells/well of 293T cells in 96-well plates following the manufacturer’s instructions. SHH protein (200 nM) and different concentrations of OLFM4 protein were added 48 h after transfection; in some assays, 1 μM cyclopamine was also added at this timepoint. GLI-reporter activity was measured 24 h later. The mean percent was obtained by comparing activity in triplicate transfections for each experimental condition to the activity for the SHH-N–treated sample (number 3; value set at 100%). Data represent the mean ± SD of triplicate experiments. ***P* < 0.01, ****P* < 0.001. Shadow triangle indicates dose of OLFM4 protein. The significance of differences between experimental groups was determined by ANOVA for (a) and (b).

**
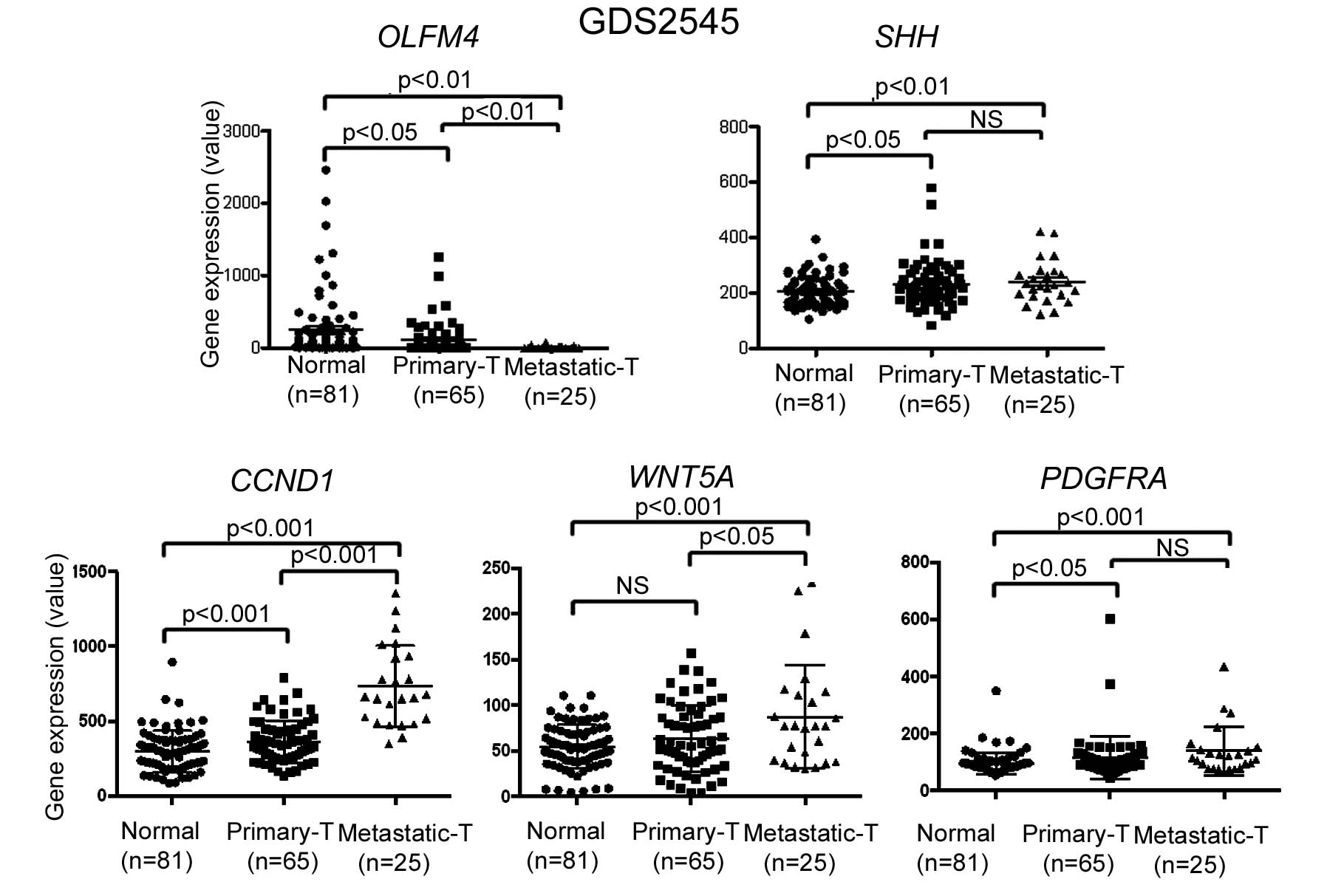
**

**Supplementary Figure S5. *OLFM4 gene* expression is decreased and *SHH* and hedgehog signaling-pathway target gene expression is increased in human prostate-cancer progression.** Gene-expression levels in published human prostate tissue GDS2545 microarray data. Scott plot graphs represent the relative expression of *OLFM4*, *SHH,* and the hedgehog signaling-pathway target genes *CCND1, WNT5A, and PDGFRA* in normal prostate, primary prostate tumors, and metastatic prostate tumors. NS, not significant. The significance of differences between any 2 stages was determined by Mann-Whitney U tests.

**Supplementary Table S1. Summary of prostatic epithelial lesions in *Olfm4* wild-type (+/+), heterozygous (+/-), and knockout (-/-) mice by age**

Genotype Total Normal Hyperplasia LG-PIN HG-PIN

+/+

3–6 months 31 31 0 0 0

10–12 months 17 16 1 0 0

18–24 months 17 15 2 0 0

+/-

3–6 months 23 19 4 0 0

10–12 months 13 8 2 2 1

18–24 months 14 9 4 1 0

-/-

3–6 months 31 20 11 0 0

10–12 months 17 4 8 5 0

18–24 months 25 1 6 11 7

LG-PIN, lower-grade prostatic intraepithelial neoplasia; HG-PIN, higher-grade prostatic intraepithelial neoplasia.

**Supplementary Table S2. The relationship of protein expression of OLFM4 and SHH in prostate-cancer tissue-array specimens with different Gleason scores**

|  |  | Gleason score | |  |  |
| --- | --- | --- | --- | --- | --- |
| OLFM4 | SHH | 4–7 | 8–10 | Total | %8–10 |
| Neg | Pos | 1 | 12 | 13 | 92% |
| Neg | Neg | 8 | 6 | 14 | 43% |
| Pos | Pos | 13 | 8 | 21 | 38% |
| Pos | Neg | 15 | 3 | 18 | 17% |
| Total |  | 37 | 29 | 66 |  |
|  |  |  |  |  |  |
| Logistic Regression for prediction of high Gleason Score | | | | | |
|  |  |  |  |  |  |
| Variable | Odds Ratio | 95% LCI | 95% UCI | P |  |
| OLFM4 Neg | 7.65 | 2.19 | 26.77 | 0.002 |  |
| SHH Pos | 5.77 | 1.66 | 20.08 | 0.006 |  |

Neg: Negative staining; Pos: Positive staining.
